# Risk Assessment of Malaria Prevalence in Ludian, Yongshan, and Jinggu Counties, Yunnan Province, after 2014 Earthquake Disaster

**DOI:** 10.4269/ajtmh.15-0624

**Published:** 2016-03-02

**Authors:** Jun Feng, Zhigui Xia, Li Zhang, Siyuan Cheng, Rubo Wang

**Affiliations:** National Institute of Parasitic Diseases, Chinese Center for Disease Control and Prevention, Shanghai, China; Key Laboratory of Parasite and Vector Biology, Ministry of Health, Shanghai, China; World Health Organization Collaborating Centre for Tropical Diseases, Shanghai, China; National Center for International Research on Tropical Diseases, Shanghai, China; GeneScience Pharmaceuticals Co., Ltd., Changchun, China

## Abstract

The objective of this study was to investigate malaria prevalence after the 2014 earthquakes in Ludian, Yongshan, and Jinggu counties, Yunnan Province, China. We collected and analyzed epidemiological data and made a risk assessment of transmission probability. From January 2005 to July 2015, 87 malaria cases were reported in the three counties, most of which (81.6%) occurred between 2005 and 2009, with five cases reported in Jinggu County between January 2014 and July 2015, of which one case was reported after the earthquake. In addition, no local transmission occurred in the three counties from 2010, and 95.5% of imported malaria occurred in patients who had returned from Myanmar. The townships of Lehong, Qingsheng, and Weiyuan were the main endemic areas in the three counties. The probability of malaria transmission in the three counties was low, but Jinggu County had a higher risk due to the existence of infected patients and an appropriate vector. With sporadic cases reported annually, close monitoring should continue to enhance early detection of a possible malaria outbreak.

## Introduction

Malaria is caused by one or more of the five species of *Plasmodium* and is transmitted via the bite of malarial female *Anopheles* mosquitoes. An estimated 198 million confirmed cases were reported from 97 countries and territories in 2013, with approximately 584,000 deaths.^1^ Natural disasters, such as earthquakes, tsunamis, and hurricanes, can result in an increased incidence of various infectious diseases.^2^ Among them, earthquakes often create conditions that lead to dramatic increases in nuisance insects and disease outbreaks.^3^ The transmission of malaria is also subject to changes in the environment due to earthquake disasters. Victims and rescue workers, living in temporary shelters, are more likely to be bitten by mosquitoes. After an earthquake in Ecuador in 1983, large-scale flooding was the primary cause of a 7-fold increase in the incidence of malaria.^4^ A total of 61 cases of malaria were diagnosed between November 2010 and February 2011 after a 7.0 magnitude earthquake in Leogane, Haiti.^5^ In addition, of all 255 patients presenting with undifferentiated fever, 76 (29.8%) were diagnosed with *Plasmodium falciparum*.^6^

On August 3, August 17, and October 7, 2014, Ludian, Yongshan, and Jinggu counties of Yunnan Province suffered devastating earthquakes of magnitudes 6.5, 5.0, and 6.6, respectively, on the Richter scale,. The earthquakes inflicted significant damage, particularly to critical infrastructure including basic utilities, transport, communication, and health care. It also caused extensive loss of human life and harm to health in the devastated area. In total, 618 deaths were reported, most of them (*N* = 617, 99.8%) from the Ludian earthquake. The warm and wet weather at the time was beneficial to insect breeding and propagation. Poor awareness of methods to control mosquito populations led to greater opportunities for mosquitoes to bite and transmit malaria. Malaria is one of the most serious public health issues in Yunnan Province. A total of 576 malaria cases were reported in Yunnan Province in 2013, comprising 14.0% of China's malaria cases for the year. The local persistence of *Plasmodium vivax* and *P. falciparum* at the China–Myanmar border has been a great challenge for malaria elimination in China. Moreover, because of the special geographical environment and complex climate conditions, mosquito species in Yunnan varies.^7^

According to the National Malaria Elimination Action Program (2010–2020) launched in 2010, based on the malaria epidemic, Ludian and Yongshan were classified as Type III counties, with Jinggu a Type II county.^8^ The main strategy for Type II counties is disposal of any possible malaria cases and active foci to interrupt local transmissions; in Type III counties, capabilities in malaria surveillance and response are emphasized to prevent malaria reintroduction.^9^ On the basis of this, close monitoring of problems in earthquake-hit areas is warranted to assess the risk of a malaria outbreak. In this context, we investigated the risk assessment of malaria prevalence of Ludian, Yongshan, and Jinggu after the 2014 earthquakes to develop a reasonable scientific monitoring and control plan to reduce economic and social harm.

## Materials and Methods

### Data collection.

We conducted a retrospective study of the period January 2005 to July 2015 to explore malaria endemic characteristics in Ludian, Yongshan, and Jinggu counties. All individual cases from the Web-Based Reporting System (WBRS) were carefully reviewed and analyzed. The data were selected by date of onset, reporting area, and final review. The WBRS parameters included species composition, geographical distribution, gender and age distribution of cases, and number of deaths. A clinically diagnosed case was defined as a patient with malaria-like symptoms but with no parasites detected by blood examination, and a laboratory-confirmed case was defined as a case confirmed using a laboratory test, including microscopy examination, rapid diagnostic tests (RDTs), or polymerase chain reaction test. Both types of cases were included in this analysis. Data on total number of cases, consisting of local infections and imported malaria cases, were obtained from another system, the Annual Reporting System.

### Risk analysis.

Through literature review and consultation of experts, qualitative analysis was conducted to determine the risk factors for malaria transmission after earthquake disasters, including the living environment of humans and mosquitoes, accessibility of malaria treatment services, presence of external rescue workers, malaria incidence, vectorial capacity for transmission of malaria by *Anopheles* mosquito, and probability of human exposure to *Anopheles* bites. After this, the risk levels for malaria after the three earthquake disasters were estimated.

### Statistical analysis.

A descriptive analysis was processed using Microsoft Excel (Microsoft Corp., Redmond, WA) and SAS software (version 9.2; SAS Institute Inc., Cary, NC). A map was created by ArcGIS 10.1 (Environmental Systems Research Institute, Inc., Redlands, CA).

## Results

**Figure 1.**
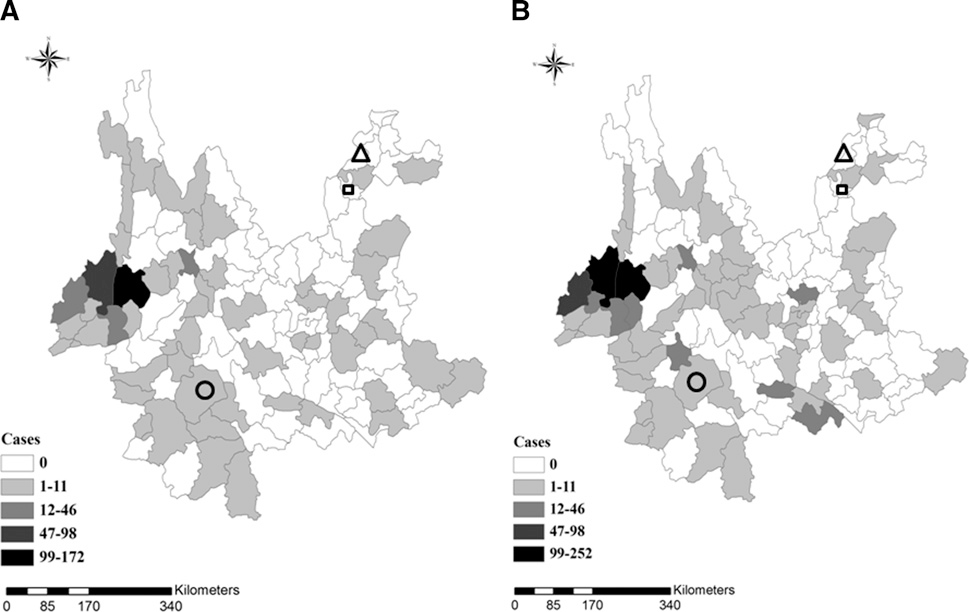
The three counties, Ludian (triangle), Yongshan (square), and Jinggu (circle), located in Yunnan Province. (**A**) Malaria cases reported from January to July 2014. (**B**) Malaria cases reported from August 2014 to July 2015.

Ludian, Yongshan, and Jinggu counties are all located in Yunnan Province, and there had always been a large number of cases in these areas (Figure 1

). A total of 87 malaria cases were recorded by WBRS in the three counties from January 2005 to July 2015, of which, cases from Ludian, Yongshan, and Jinggu represent 11.5% (*N* = 10), 34.5% (*N* = 30), and 54.0% (*N* = 47), respectively. Of all 87 cases, 71 (81.6%), were reported from 2005 to 2009. The cases recorded between January 2014 and July 2015 were seen only in Jinggu County (*N* = 5), and this is significantly different from the period 2005 to 2013. No malaria cases occurred in Ludian and Yongshan counties during or after 2010 and 2012, respectively. *Plasmodium vivax* was the major species (*N* = 73) in the three counties, while *P. falciparum* was reported in five cases, four of which occurred in Jinggu County. In addition, one *P. falciparum* death case was seen in Ludian County in 2005.

Most cases (86.2%) occurred in males (*N* = 75), with 13.8% in females (*N* = 12). Malaria cases were distributed across ages ranging from 10 to 59 years, with the highest malaria cases converged on the group aged 20–39 years.

### Regional distribution.

**Figure 2.**
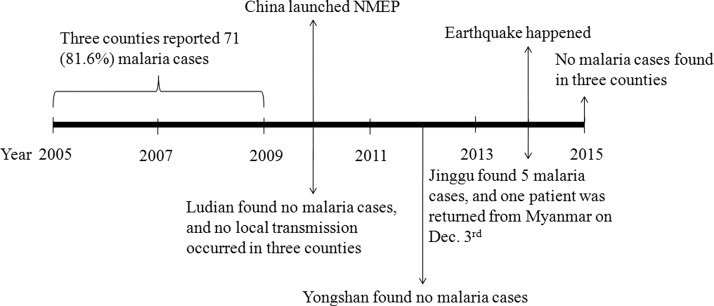
Scheme of malaria epidemiological characteristics of three counties of Yunnan Province, January 2005 to July 2015. Only Jinggu County reported a malaria case after the earthquake, on December 3, 2014, and this patient had returned from Myanmar.

Between 2005 and 2013, the townships of Lehong (*N* = 6, 60%, 6/10), Qingsheng (*N* = 20, 66.7%, 20/30), and Weiyuan (*N* = 21, 44.7%, 21/47) were the main endemic areas in Ludian, Yongshan, and Jinggu counties, respectively. From January 2014 to July 2015, no malaria cases occurred in the townships of Lehong and Qingsheng, whereas four cases were found in Weiyuan; one of these was an individual who had returned from Myanmar and was reported on December 3, 2014, nearly 2 months after the earthquake (Figure 2

).

### Source of infection.

The Annual Reporting System data indicated that between January 2005 and July 2015, 43 cases were locally acquired (49.4%) and 44 cases were imported (50.6%). No local transmission was found in the three counties from 2010. However, it is noted that 15 domestic cross-regional cases were reported, with 13 of them, from Hainan Province in 2007 and 2008, occurring in Yongshan (*N* = 12) and Ludian counties (*N* = 1). The other two patients were from Baoshan (*N* = 1) and Canyuan counties (*N* = 1) in Yunnan Province. For the imported cases, 42 (95.5%, 42/44) patients had returned from Myanmar, and the other two patients returned from Laos and Angola. From January 2014 to July 2015, five imported cases occurred in Jinggu County, all in individuals who had returned from Myanmar (Figure 2).

### Interval between onset and diagnosis.

From January 2005 to July 2015, the time interval between symptom onset and diagnosis was revealed to be an average of 19.7, 16.0, and 11.6 days for Ludian, Yongshan, and Jinggu, respectively, and all these intervals were greater than the national average interval (7.7 days). In 2014, the average interval between symptom onset and diagnosis in Jinggu County was 18.0 days, with one patient, initially diagnosed with *P. vivax*, reclassified as *P. falciparum* after 28 days following confirmation in the provincial reference laboratory. The symptom onset-diagnosis interval of the single case reported on December 3, 2014 after the earthquake was 1 day.

### Risk analysis.

In the three earthquake-affected counties, buildings, transportation, communication, and health systems were destroyed, leading to a change in the living environment of humans and mosquito habitat. Many people lived in tents or other temporary shelters outside houses, and they do not care about using personal protections such as nets and mosquito incense against mosquito bites, providing greater opportunity for mosquito bites than before the earthquakes. The houses and infrastructure in Ludian County were the most seriously damaged, with exposure to mosquito bites higher than in the other two counties.

Health systems may not have been seriously affected and may have been even better than before in Yongshan and Jinggu counties. Health infrastructure in Ludian County was seriously damaged, with health service accessibility decreased. Many rescue workers from outside the earthquake disaster area participated in rescue activities in the three counties. When people from other areas of high malaria incidence visited disaster areas, there is the possibility of malaria being imported into these areas.

Ludian and Yongshan counties observed no malaria cases from 2010 and 2012, respectively. But in Jinggu County, malaria cases were reported in 2014. Historical *Anopheles* survey data were lacking in Ludian and Yongshan counties. *Anopheles sinensis*, considered an important vector of *P. vivax* malaria in China because of its wide distribution and high density,^10^ is also the major mosquito species (75.4% of total mosquitoes) in Jinggu County, according to mosquito surveys from 2007 to 2012.^11^

On the basis of the risk factor analysis, the probability of malaria transmission in all three counties was low. The presence of malaria cases and vectors for transmission are the basic conditions for malaria transmission. There was higher risk of malaria transmission in Jinggu County than in the other two counties after the earthquake disaster (Table 1).

## Discussion

Animal and vector habitats are hugely changed after an earthquake. Because of subsequent poor living conditions, increased exposure to mosquitoes may increase the transmission risk of vector-borne diseases such as malaria, dengue fever, and plague.^12^,^13^ Furthermore, rainfall or overflow of rivers can create new breeding sites, resulting in an increase in vector population and potential for malaria transmission. An earthquake in Costa Rica's Atlantic region in 1991 was associated with changes in habitat that were beneficial for breeding and preceded an extreme rise in the number of malaria cases.^14^ The crowding of infected and susceptible hosts, a weakened public health infrastructure, and the interruptions of ongoing control programs are the risk factors for vector-borne disease transmission.^15^ Ludian, Yongshan, and Jinggu counties suffered serious earthquake damage in 2014. These three counties have a subtropical climate that is warm, wet, and rich in insects. As the earthquakes occurred in the transmission season, mosquito populations typically increased. Also, many houses were damaged by the earthquake, a factor that increased the chance of humans receiving an insect bite.

Malaria is one of the most widespread infectious diseases transmitted by *Anopheles* mosquitoes.^16^ China has succeeded in preventing and controlling malaria in the past 10 years with a significant decline in the number of cases, especially locally acquired cases.^17^ This pattern was also seen in the three counties with locally acquired cases absent since 2010; theoretically all three counties had entered into the elimination stage with no locally acquired cases reported in the last 3 years.^18^

From January 2005 to July 2015, 87 malaria cases were recorded in the three counties, with cases in Jinggu County accounting for 54% of this total, accordingly Jinggu County was classified as a Type II county in 2010.^8^ Although locally acquired cases declined, it still persist in the border counties in Yunnan Province; in 2014, 47 local cases were reported in the nine counties at the China–Myanmar border, accounting for 82.5% of total locally acquired cases throughout the country. Population movement, including people returning from other regions of the country to aid disaster-affected areas, could potentially contribute to the reemergence of malaria.^19^

On the other hand, the vector for transmission continued to persist in the local area. For example, in Jinggu County, 3,753 *An. sinensis* were found between 2007 and 2012, playing the dominant role (75.4%) in local vector species, with peak development between April and October.^11^ Imported malaria cases, whether domestically cross-regional or returned from other endemic countries, may facilitate high risks to those counties in which *Anopheles* mosquitoes are prevalent during the transmission season and pose a great challenge to malaria elimination. Therefore, personal protection is important during disaster situations, and Centers for Disease Control and Prevention should make mosquito repellents available to all affected populations, encourage the use of insecticide-treated mosquito nets, and carry out the impregnation of all material for temporary shelter (such as tents) with insecticides.

Poor access to health services is another significant risk factor that can lead to delayed diagnosis.^20^ This was reflected in our findings; the interval of onset of symptoms to diagnosis in the three counties was fairly lengthy. This may have been due to the occurrence of relatively few recent cases, resulting in a gradual loss of diagnosis awareness by medical staff. Early diagnosis and treatment of malaria patients is crucial for saving lives, as delayed diagnosis enhances transmission risks at the border such as China–Myanmar border because the *Anopheles* mosquito was detected in this area. The Haiti earthquake disaster of 2010 provides an example of the detection of malaria and adoption of appropriate treatment after an earthquake disaster. Although malaria RDTs were not a regular part of the national malaria control strategy in Haiti, the Haitian Ministry of Health and Population allowed the use of malaria RDTs during the 90-day period immediately after the earthquake, from January 12 to April 12, 2010. A total of 317 (20.3%) patients were positive among 1,564 tested patients indicating medical teams trained in the use of malaria RDTs had high testing rates for suspected malaria cases.^21^

After the Ludian, Yongshan, and Jinggu earthquakes, the Health and Family Planning Commission of Yunnan Province, along with Ludian, Yongshan, and Jinggu Health Disease Control departments organized health and epidemic prevention teams and initiated epidemic prevention measures by providing full sanitation coverage and controlling disease vectors. These teams conducted disinfection in key areas such as temporary residences for victims and relief workers, and reached into hard-hit communities and rural villages to conduct immunization and prevention work. In the first month after the earthquakes, areas of more than 50,000, 35,000, and 40,000 m^2^ were disinfected in Ludian, Yongshan, and Jinggu counties, respectively.

Ludian, Yongshan, and Jinggu counties were hit hard by the earthquakes, with direct economic losses estimated at US$1.02 billion, US$24 million, and US$274 million, respectively. Ludian was affected the most due to higher population density and poorer economic and health conditions. Although the probability of malaria transmission in the three counties was low after the earthquakes, malaria surveillance was enhanced because of the existence of transmission vectors. The government and health departments collaboratively took effective measures to prevent infectious disease epidemics after the disaster. Indoor residual spraying of insecticides in the affected areas may have prevented the outbreak of malaria. Further, early detection of a possible malaria outbreak can be enhanced by monitoring daily case numbers through WBRS. No locally acquired malaria cases occurred in the three counties after the earthquakes.

## Conclusions

Earthquakes occur frequently in Yunnan Province, especially in the border area between China and Myanmar, where local malaria still exists. Malaria transmission risk should be assessed timeously and continually after earthquakes. On the basis of the risk assessment results, emergency measures should be timely and evidence based, using malaria case detection and treatment, mosquito surveys, disinfection, and so on to avoid an outbreak of malaria after an earthquake^22^ and contributing to the goal of malaria elimination in China by 2020. Also other countries nearing elimination should also review disaster response guidelines to consider malaria risk and its management in these environments.

## Figures and Tables

**Table 1:** Risk assessment of three counties

Indicators	Ludian County	Yongshan County	Jinggu County
Malaria cases			
Before earthquake	10	30	46
After earthquake	0	0	1
Most recent local transmission (year)	2003	2003	2009
Vector survey	N/A	N/A	*Anopheles sinensis* (75.4%)
Total population	43.5 million	42.0 million	30.6 million
Major source of imported cases	Domestic (Hainan Province)	Domestic (Hainan Province)	Myanmar
Risk level	Relatively low	Relatively low	Low

N/A = not applicable.
